# Quantitative Structure Activity Relationship Studies and Molecular Dynamics Simulations of 2-(Aryloxyacetyl)cyclohexane-1,3-Diones Derivatives as 4-Hydroxyphenylpyruvate Dioxygenase Inhibitors

**DOI:** 10.3389/fchem.2019.00556

**Published:** 2019-08-20

**Authors:** Ying Fu, Yong-Xuan Liu, Ke-Han Yi, Ming-Qiang Li, Jia-Zhong Li, Fei Ye

**Affiliations:** ^1^Department of Applied Chemistry, College of Science, Northeast Agricultural University, Harbin, China; ^2^School of Pharmacy, Lanzhou University, Lanzhou, China

**Keywords:** 4-hydroxyphenylpyruvate dioxygenase inhibitors, three-dimensional quantitative structure activity relationship, molecular docking, molecular dynamics, molecular mechanics Poisson–Boltzmann surface area

## Abstract

4-Hydroxyphenylpyruvate dioxygenase (HPPD) is a significant enzyme in the biosynthesis of plastoquinone and tocopherol. Moreover, it is also a potential target to develop new herbicide. The technology of computer-aided drug design (CADD) is a useful tool in the efficient discovery of new HPPD inhibitors. Forty-three compounds with known activities were used to generate comparative molecular field analysis (CoMFA) and comparative molecular similarity indices analysis (CoMSIA) models based on common framework and molecular docking. The structural contribution to the activity was determined, which provided further information for the design of novel inhibitors. Molecular docking was used to explain the changes in activity caused by the binding mode between ligand and protein. The molecular dynamics (MD) results indicated that the electrostatic energy was the major driving force for ligand–protein interaction and the Phe403 made the greatest contribution to the binding. The present work has provided useful information for the rational design of novel HPPD inhibitors with improved activity.

## Introduction

4-Hydroxyphenylpyruvate dioxygenase (HPPD), a Fe(II)-dependent non-heme oxygenase, belongs to the α-ketoacid family and plays different roles in organism and plant cells (Rocaboy-Faquet et al., 2014; Huang et al., 2016). It catalyzes the conversion of 4-hydroxyphenylpyruvate (HPPA) into homogentisate (HGA), which is the first committed metabolism of the tyrosine catabolism pathway in humans (Raspail et al., 2011; Moran, 2014; Silva et al., 2015). In plants, HPPD is an essential element in the biosynthesis of plastoquinone and tocopherol; both of them are significant cofactors in the photosynthesis. Inhibition of HPPD will lead to a deficiency of the isoprenoid redox cofactors, followed by the presence of bleaching in plants, eventually bringing necrosis and death (Zou et al., 2007; Wang et al., 2015a).

HPPD has been the subject as an important target for development of new herbicides and multiple series of compounds have been designed and synthesized (Wang et al., 2014, 2015b; Ndikuryayo et al., 2019). When applied pre- or post-emergence, HPPD inhibitors provide control to the important broad leaf weeds in maize and a certain amount of annual weeds (López-Ramos and Perruccio, 2010). HPPD inhibitor herbicides manifest many advantages, for instance, good activity, broad-spectrum weed control, low mammalian toxicity, low residual rate, desired selectivity, and environment friendly (Beaudegnies et al., 2009; Cho et al., 2013; Schultz et al., 2015). However, the first case of HPPD inhibitor herbicide resistance was confirmed in Iowa and Illinois simultaneously in 2010 (Hausman et al., 2011; Kohlhase et al., 2018). Monoculture production systems and multiple uses of herbicides with similar mechanism of action contributed to the generation of weeds resistance to the existing HPPD herbicides (Duke, 2012; Larran et al., 2017; Ye et al., 2018). In response to the evolution of herbicide resistance in weeds, discovering novel inhibitors with high efficiency is urgent. Triketone compounds represent one of the HPPD herbicides, and its substructure is typically based on the 2-benzoyl or 2-heteroaroyl cyclohexane-1,3-dione (Matringe et al., 2005; Roy and Paul, 2010). The activity of triketone HPPD inhibitor was better than any other categories, and they can directly exert effects in the weeds, causing plants to die (Ndikuryayo et al., 2017; Lin et al., 2019).

**Figure 1:**
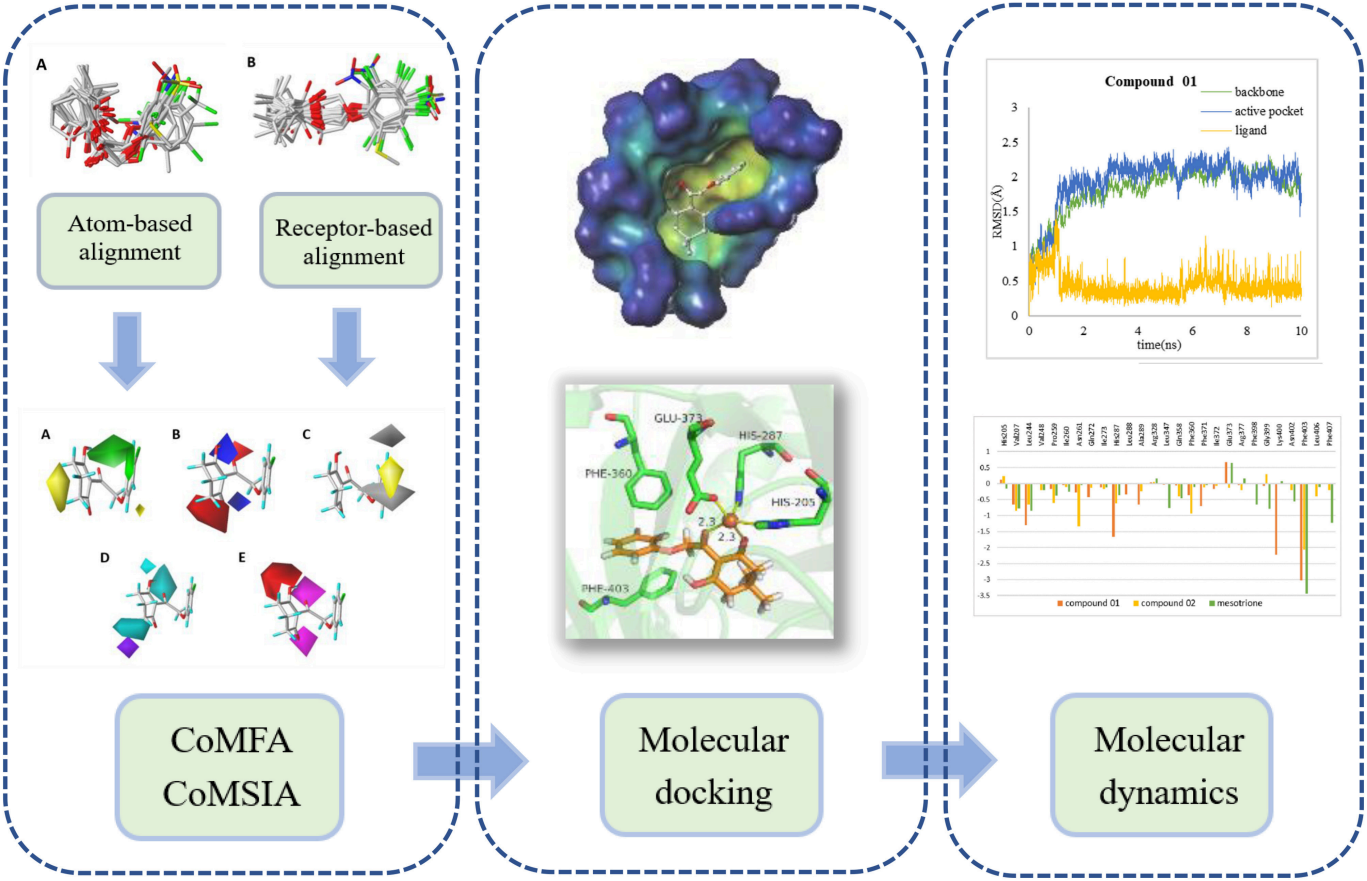
The computational workflow was applied.

In this research, a series of 2-(aryloxyacetyl)cyclohexane-1,3-diones derivatives were selected to establish three-dimensional quantitative structure activity relationship (3D-QSAR), applying comparative molecular field analysis (CoMFA) and comparative molecular similarity indices analysis (CoMSIA). Subsequently, molecular docking and molecular dynamics (MD) study was applied to analyze the robustness of the ligands inside the receptor cavity and to learn more about the binding interactions. The analysis strategy is shown in Figure 1. The obtained information will contribute to the rational design of novel HPPD inhibitors with powerful activity in the future.

## Materials and Methods

### Data Collection and Preparation

A total of 43 2-(aryloxyacetyl)cyclohexane-1,3-diones derivatives as effective inhibitors were collected to build 3D-QSAR models based on the published literature (Wang D.W. et al., 2016). The activity range of the inhibitors was 0.029–5.571 μM. The structures of these compounds were built and optimized by SYBYL 6.9 to generate 3D structures with appropriate conformation (SYBYL, 2006; Arvind et al., 2014). Simulations were carried out by employing Tripos force field with energy termination of 0.005 kcal/mol, and a maximum of 1,000 iterations. Gasteiger-Hückel charges were used to calculate the partial atomic charges (Zhang et al., 2010).

### Molecular Docking

**Figure 2:**
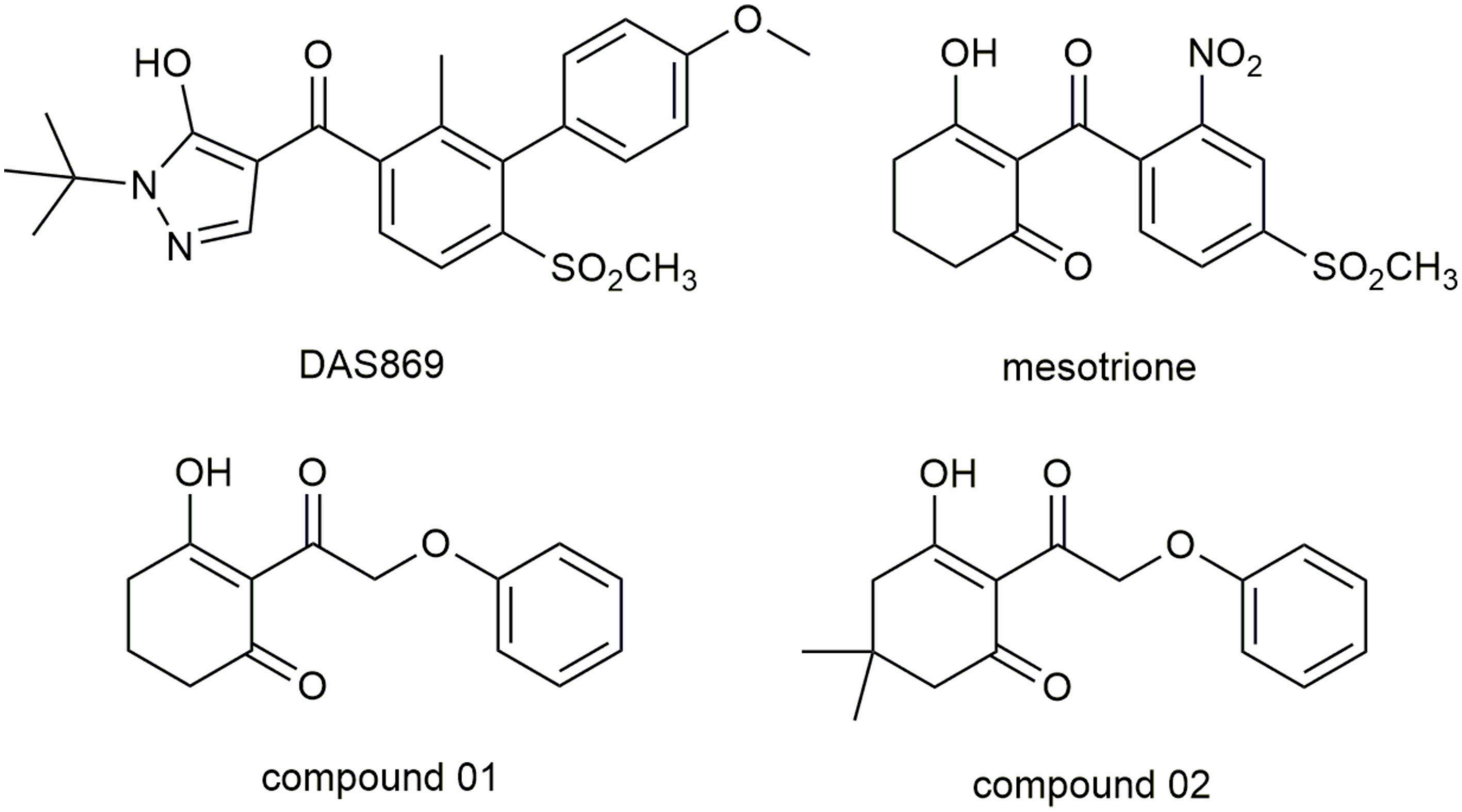
The chemical structure of DAS869, mesotrione, and compounds 01 and 02.

Molecular docking study was applied to obtain corresponding active conformations and analyze receptor–ligand interaction. During the docking operation, 43 HPPD inhibitors were docked into the active pocket of *Arabidopsis thaliana* HPPD (*At*HPPD) using the Accelrys Discovery Studio v3.5 (Catalyst, 2005). The x-ray crystal structure of the *At*HPPD (PDB code: 1TFZ) was obtained from the RCSB Protein Data Bank (Yang et al., 2004). All the redundant water molecules and co-crystallized ligand were deleted from the complex before docking study; hydrogen was added to the protein (Yang et al., 2013; Wang D.W. et al., 2016). CHARMm force field was added to the receptor and ligands, and the binding site was defined from the known ligand pose (Fu et al., 2019a,b). Docking operation was performed by CDOCKER protocol with the default docking setting, in which 10 conformations were saved about each ligand based on docking score values (Wu et al., 2003). The postures of the ligands were checked manually, comparing with the co-crystallized ligand (DAS869) in the 1TFZ and other reported inhibitor-enzyme complex crystals (Lin et al., 2019). The chemical structure of the DAS869 is shown in Figure 2. Molecules removed the unreasonable conformations and were used to build the QSAR models. The ligands with the best CDOCKER_ENERGY were employed for the analysis of the binding mode.

### Alignment of Compounds

Alignment step was extremely important in the process of the development of 3D-QSAR models. The whole data set was divided into training set and test set to develop and validate the model. Random selection is a popular utilized method to build the QSAR models, and the diversity of chemical structures and activities was also considered. Nine compounds were selected as test set, and their structures were abundant, while their p*K*_i_ values were uniformly distributed in terms of the value range of the whole set.

To develop an ideal model, two different alignment measures were employed. The first alignment rule was a common framework approach, which appointed the molecule 12 with the best activity as the framework template (Wang J.H. et al., 2016). In this strategy, a multi-search method was applied to search aligning postures with the lowest energy, followed by using the “align database” tool in SYBYL 6.9; all the other compounds were superimposed to the template with the form of common scaffold. Differing from the previous protocol, the second strategy was a receptor-based approach, which states that all molecular conformations were obtained from docking simulation rather than the previous one on the basis of atoms. The best active molecule 12 with docking conformation was chosen as the template molecule.

### 3D-QSAR Model Generation and Validation

A standard development of CoMFA or CoMSIA model was performed by the partial least squares (PLS) regression analysis to select interrelated components and set up the optimal 3D-QSAR model (Dong et al., 2017). A sp3 carbon atom, as the steric probe, was used with a charge of +1.0 in the process of steric and electrostatic field in CoMFA generation. For CoMSIA analysis, five descriptor fields, namely steric, electrostatic, hydrophobic, H-bond donor, and H-bond acceptor field, were selected to simulate models (Kothandan et al., 2011). p*K*_i_ values, which were negative logarithm converted from *At*HPPD inhibition *K*_i_ values, were carried out as the dependent variable for model development. To establish a model with excellent prediction ability, the leave-one-out (LOO) strategy was used to carry out the cross-validation analysis. The optimum number of components (ONC) was calculated and the cross-validated coefficient (*Q*^2^) was obtained to evaluate the model. The model was followed by the non-cross-validation analysis and the coefficient of determination (*R*^2^), the standard error of estimate (SEE), and the F value were calculated based on the ONC originated from LOO (Arvind et al., 2014). The predicted correlation coefficient for the test set ($R_{pred}^{2}$) was used to examine the predictive power of the model. In addition, the reliability and effectiveness of the model were measured through comparing the experimental p*K*_i_ values with the predicted p*K*_i_ values of the data set.

### Molecular Dynamics

A portion of docking complex adopted for MD was designed to explore the major driving force for ligand–receptor interactions and analyze the related amino acid residues. Two representative inhibitors, compound 01 and 02, and commercial herbicide, mesotrione, were selected to perform simulation in the best pose of docking. The chemical structure of these three compounds is shown in Figure 2. Compound 01 represented the backbone of this class of compounds, and the change in the activity of compound 02 was attributed to the introduction of methyl groups at the 5-position of R_1_. Mesotrione, a widely used herbicide, was used as a control compound in this study.

The ligand, receptor, and complex information of the two docking structures were introduced in Amber 16 (Case et al., 2016). Initially, the ligands were formatted in Antechamber program and AM1-BCC protocol was employed to calculate the partial atomic charges of molecules. The metal ion, Fe(II), in the protein needed to be specially treated, which was critical to build non-bonding model with simple form and excellent transferability implemented. The metal center parameter builder (MCPB) module of Amber was used to modify Fe(II)-amino acids interaction including His205, His287, and Glu373 (Peters et al., 2010; Li, 2014). The side chain connecting Fe(II) was treated by the restrained electrostatic potential (RESP) tool of Gaussian03. Meanwhile, the atomic partial charges and the geometry optimization were calculated (Frisch et al., 2004). Angle, bond, torsion, improper torsion, van der Waals, and other information parameters were performed through the MCPB. The charge neutralized and solvated progress were generated in the “LEaP” module. In order to produce the appropriate topologic and coordinate files required for the MD simulations, the generalized Amber force field gaff and ff14SB force field were used for ligand and receptor, respectively (Hornak et al., 2006). A rectangular box of TIP3P water was added to the system with a boundary of 10 Å from the edge of the box to the complex atom, and sodium ions that assisted to maintain the electrical properties reflected the neutral state (Gadd et al., 2017). The optimization process was divided into three parts with different constraints. Each section included the steepest descent method of 2,500 steps and the conjugate gradient method of 2,500 steps as well. Heating of the system was a gentle rise in temperature from 0 to 298 K in the canonical (NVT) ensemble with 20 kcal mol^−1^ Å^−2^. A density balance achieved in 500 ps with fixed protein backbone atoms to allow relaxation of the solvent and overall equilibrium lasting 1 ns was performed to ensure the equilibrium state of the MD simulation conditions. Subsequently, the whole simulation was over the course of 10 ns with a 2-fs step.

The procedure of combining free energy calculation was applied to the molecular mechanics method based on all atoms and Poisson-Boltzmann solvation area (MM-PBSA) measure (Hao et al., 2011). The average over the extracted snapshots from the MD stable trajectories was used to compute the free energy. Based on the following equations, the correlative binding free energies were obtained:

(1) $$\begin{array}{r} \Delta G_{bind}=G_{cpx}-G_{rec}-G_{lig} \end{array}$$

(2) $$\begin{array}{r} \Delta G_{bind}=\Delta E_{MM}+\Delta G_{sol}-T\Delta S \end{array}$$

(3) $$\begin{array}{r} \Delta E_{MM}=\Delta E_{int}+E_{ele}+E_{vdw} \end{array}$$

(4) $$\begin{array}{r} \Delta G_{sol}=\Delta G_{PB}+\Delta G_{SA} \end{array}$$

where Δ*E*_MM_ is determined by the internal energy (Δ*E*_int_) contributed from bonds, angles, and torsions, the van der Waals energy (Δ*E*_vdw_), and electrostatic force (Δ*E*_ele_). ΔGsol denotes the solvation free energy, which consists of the polar solvation contribution (Δ*G*_PB_) and non-polar solvation contribution (Δ*G*_SA_). As the contribution of entropy is insignificant for a series of similar systems, *T*Δ*S* items are excluded in our study (Fu et al., 2017, 2018).

To obtain the detailed interactions between the HPPD and inhibitors, the binding free energy was decomposed onto each individual residue using the MMPBSA.py module. In the decomposition process, the van der Waals contribution (Δ*E*_vdw_), the electrostatic contribution (Δ*E*_ele_), and the free energy of solvation (Δ*G*_sol_) in the binding process of enzyme and ligands were calculated and the contribution of entropy was omitted.

(5) $$\Delta G_{\text{inhibitor}\_\text{residue}}=\Delta E_{\text{vdw}}+\Delta E_{\text{ele}}+\Delta G_{\text{sol}}$$

## Results and Discussion

### 3D-QSAR Models

The framework of molecules, each molecular structure, and the activity values are shown in Table 1. Six statistical parameters including the *Q*^2^, ONC, *R*^2^, SEE, *F*, and $R_{pred}^{2}$ value are obtained to assess the creditability of each 3D-QSAR model. As far as *Q*^2^ and *R*^2^ are concerned, they are considered as two vital standards to evaluate the quality and predictive capability of the QSAR models. In addition, a low SEE value and good *F* and $R_{pred}^{2}$ values are also crucial for a reliable model.

**Table 1:** The structure of 2-(aryloxyacetyl)cyclohexane-1,3-diones derivatives and corresponding experimental and predicted activity.

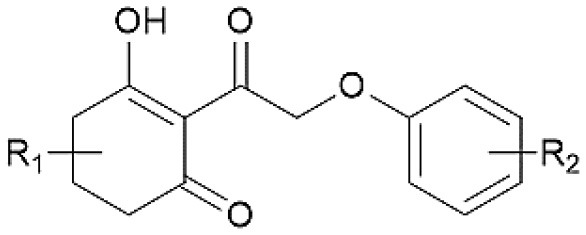							
**Comp**.	**R**_**1**_	**R**_**2**_	**p*****K***_**i**_				
			**Exp**.	**Common framework**		**Docking conformation**	
				**CoMFA**	**CoMSIA**	**CoMFA**	**CoMSIA**
01	H	H	5.906	5.923	5.954	5.962	6.007
02	5,5-diCH_3_	H	5.254	5.257	5.218	5.243	5.218
03	H	2-CH_3_	6.049	6.067	6.052	6.061	5.996
04	5,5-diCH_3_	2-CH_3_	5.797	5.806	5.810	5.774	5.774
05^*^	H	3-CH_3_	6.206	6.266	6.236	6.443	6.643
06	H	4-CH_3_	6.005	5.979	5.992	5.969	6.005
07	H	4-OCH_3_	6.521	6.496	6.534	6.532	6.557
08	H	2-SCH_3_	6.223	6.218	6.242	6.237	6.216
09^*^	H	2-Cl	5.942	6.102	6.115	5.634	5.561
10	5,5-diCH_3_	2-Cl	5.669	5.685	5.614	5.683	5.677
11	H	3-Cl	7.125	7.105	7.078	7.042	7.142
12	H	4-Cl	7.538	7.549	7.403	7.568	7.426
13	H	2-CF_3_	6.873	6.899	6.826	6.862	6.894
14^*^	H	3-CF_3_	6.251	6.383	6.185	6.412	6.187
15	H	4-CF_3_	5.978	5.978	6.049	5.981	6.019
16	H	2-NO_2_	7.347	7.338	7.316	7.344	7.328
17	H	4-SO_2_CH_3_	6.451	6.448	6.404	6.481	6.419
18^*^	5-CH_3_	4-SO_2_CH_3_	6.264	6.126	6.226	6.171	6.202
19	H	2,3-diCl	6.947	6.939	7.006	6.959	6.949
20	H	2,4-diCl	7.367	7.419	7.204	7.351	7.314
21	H	2,5-diCl	6.917	6.895	6.976	6.954	6.884
22^*^	H	2,6-diCl	7.347	7.298	7.302	7.252	7.194
23	H	3,4-diCl	6.573	6.562	6.553	6.576	6.624
24	H	3,5-diCl	6.020	6.021	6.018	6.047	5.987
25	5-CH_3_	2,4-diCl	6.706	6.687	6.708	6.728	6.723
26^*^	5,5-diCH_3_	2,4-diCl	5.318	5.154	5.254	5.123	5.788
27	4,4-diCH_3_	2,4-diCl	6.616	6.599	6.576	6.629	6.628
28	H	2,4-diBr	7.180	7.180	7.209	7.158	7.204
29	5,5-diCH_3_	2,4-diBr	7.056	7.059	7.057	7.062	7.022
30	H	2-CH_3_-4-F	6.682	6.693	6.773	6.701	6.64
31	H	2-CH_3_-4-Cl	7.509	7.487	7.387	6.933	6.468
32	5-CH_3_	2-CH_3_-4-Cl	7.432	7.386	7.476	7.451	7.493
33	5,5-diCH_3_	2-CH_3_-4-Cl	6.959	6.965	6.935	6.932	6.932
34^*^	4,4-diCH_3_	2-CH_3_-4-Cl	5.699	5.612	5.715	5.375	5.364
35	H	2-CH_3_-4-Br	7.108	7.154	7.229	7.081	7.164
36	H	2-CH_3_-4-NO_2_	7.114	7.130	7.145	7.093	7.113
37	H	2-Cl-4-F	6.262	6.278	6.237	6.212	6.28
38^*^	H	2-Cl-4-NO_2_	7.469	7.580	7.536	7.074	7.478
39	H	2-F-4-Cl	6.455	6.439	6.516	6.428	6.438
40	H	2-NO_2_-3-CH_3_	7.161	7.149	7.166	7.197	7.185
41	H	3,5-diF-4-CN	7.086	7.076	7.116	7.096	7.091
42	H	2,4,6-tri-Cl	6.735	6.736	6.725	6.722	6.765
43^*^	H	2,3,4,5,6-5F	7.260	7.163	7.189	7.155	7.411
Mesotrione			7.886				

* *Indicates the test set of compounds*.

The parameters of the obtained models are listed in Table 2. The best CoMFA model based on common framework was established with a best cross-validated correlation coefficient value (*Q*^2^ = 0.872) and a high conventional correlation coefficient (*R*^2^ = 0.999). The optimum number of components (ONC) was 10 and the contributions of steric and electrostatic fields were 52.3 and 47.7%, respectively. The standard error of estimate (SEE) was 0.024, the *F* value was 1776.949, and the predicted correlation coefficient ($R_{pred}^{2}$) was 0.863, which proved that the model possessed great predictable capability. The CoMFA model based on molecular docking was built with *Q*^2^ = 0.693 and *R*^2^ = 0.998, and at this time, the ONC value was 10; the SEE value of 0.034, the *F* value of 898.323, and the $R_{pred}^{2}$ value of 0.828 were also obtained. The contribution rate of the steric field was 83.4% and that of the electrostatic field was 16.6%, which suggested that the steric field had more influence on the inhibition than the electrostatic field. The statistical values of the CoMFA model from molecular docking were found to be inferior to those from the common framework, especially on the cross-validated correlation coefficient value (0.693 and 0.872, respectively).

**Table 2:** Results of CoMFA and CoMSIA models.

	**Common framework**		**Docking conformation**	
**Parameter**	**CoMFA**	**CoMSIA**	**CoMFA**	**CoMSIA**
*Q^2^*	0.872	0.864	0.693	0.823
ONC	10	10	10	10
*R^2^*	0.999	0.990	0.998	0.995
SEE	0.024	0.069	0.034	0.050
*F*	1776.949	215.356	898.323	425.569
$R_{pred}^{2}$*	0.863	0.850	0.828	0.801
**Contribution (%)**				
Steric	52.3	9.4	83.4	9.7
Electrostatic	47.7	24.2	16.6	16.4
Hydrophobic	–	28.5	–	31.4
Donor	–	26.5	–	30.6
Acceptor	–	11.3	–	11.9

* *Indicates the statistical characteristics for the test set*.

The CoMSIA model, based on common framework, gave a good *Q*^2^ value of 0.864 and an ideal *R*^2^ value of 0.990 with 10 components. All the parameters of the model, containing the SEE value of 0.069, the *F* value of 215.356, and the $R_{pred}^{2}$ value of 0.850, are shown in Table 2. The model was generated through a combined use of five fields, steric field, electrostatic field, hydrophobic field, hydrogen bond donor, and hydrogen bond acceptor. The contributions were 9.4, 24.2, 28.5, 26.5, and 11.3%, respectively. Electrostatic, hydrophobic, and hydrogen bond donor field had a greater impact on the CoMSIA results, and by modifying these characteristics, the activity would be promoted. The CoMSIA model provided more comprehensive information than CoMFA. The CoMSIA model based on molecular docking led to a satisfactory *Q*^2^ value of 0.823 using 10 components and an *R*^2^ value of 0.995 with SEE = 0.050, *F* = 425.569, and $R_{pred}^{2}$ = 0.801. The contributions of steric field, electrostatic field, hydrophobic field, hydrogen bond donor, and hydrogen bond acceptor were 9.7, 16.4, 31.4, 30.6, and 11.9%, respectively, which was roughly similar as the proportion of the CoMSIA model from the common framework. The parameters indicated that the CoMSIA models generated by two strategies had both satisfying conventional statistical correlation and good predictive ability of bioactivity.

**Figure 3:**
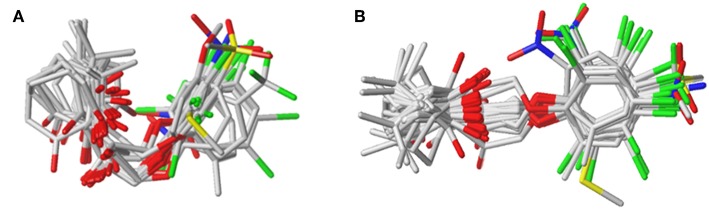
The alignment of the molecules using **(A)** the common framework and **(B)** the docking simulation. Molecules are displayed in white for common C, red for O, blue for N, yellow for S, and green for F and Cl atoms, respectively. For a clear observation, hydrogen atoms are hidden.

**Figure 4:**
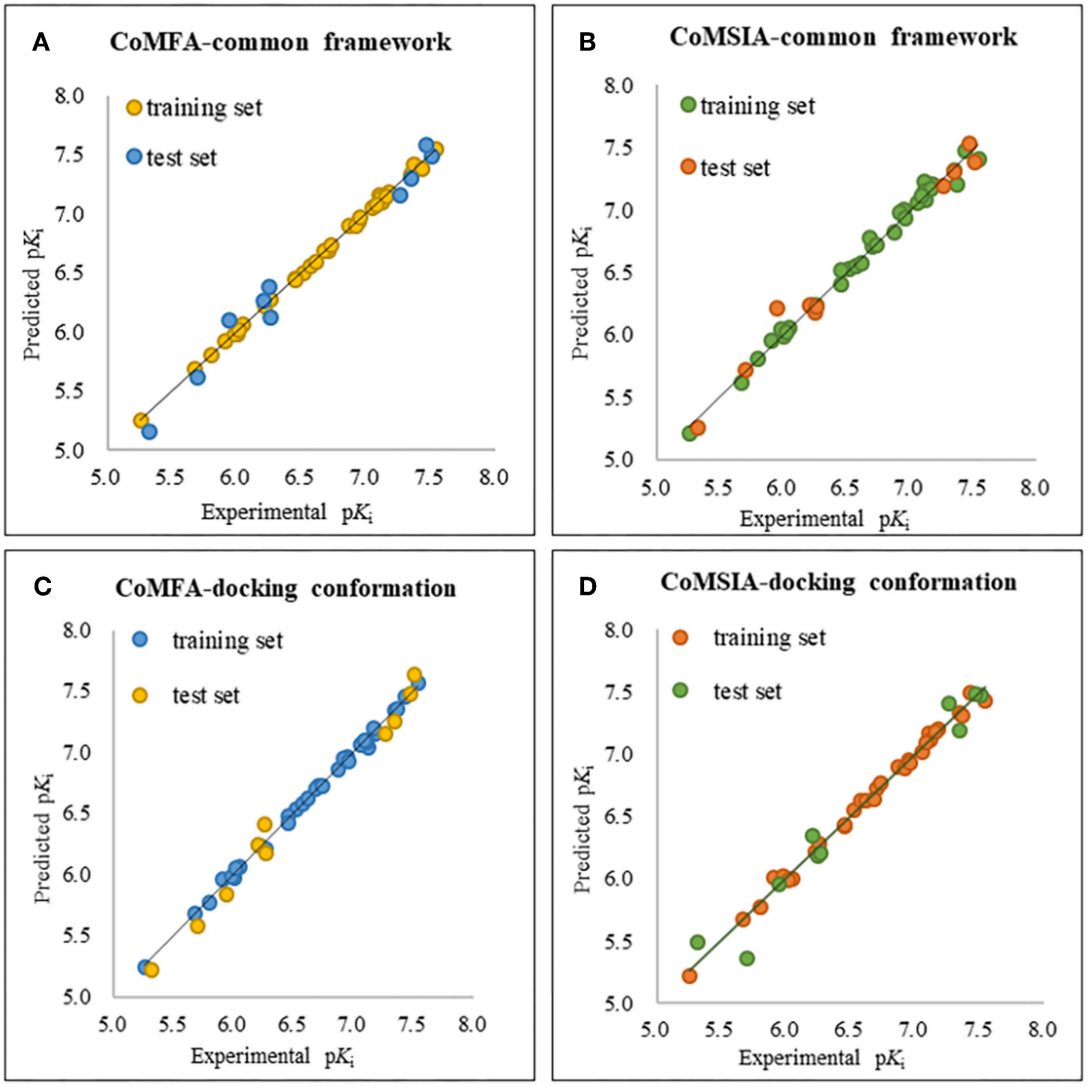
The plot of experimental and predicted activity based on the common framework **(A,B)** and molecular docking **(C,D)**.

The plots of the experimental vs. the predicted activity values for all of the compounds are shown in Figure 3. The linear relation between the experimental and predicted p*K*_i_ was excellent for either the CoMFA or CoMSIA model from the common framework or molecular docking, indicating closeness of the experimental and predicted biological activity values, and the strong predictive power of the model could be verified. The alignments of the molecules are shown in Figure 4. The molecules used for the common framework alignment were derived from multiple search, which resulted in the conformation of the molecule exhibiting a low energy fold (Figure 4A). The alignment from molecular docking was not the same. During the interaction of the protein and the inhibitors, the conformations of the molecules were in a stretched state (Figure 4B). The conformation of the oxygen atoms used for chelation was highly similar, resulting in a high degree of overlap in this portion. The remaining molecular groups exhibit different postures under the influence of proteins due to their different properties.

### CoMFA Results

**Figure 5:**
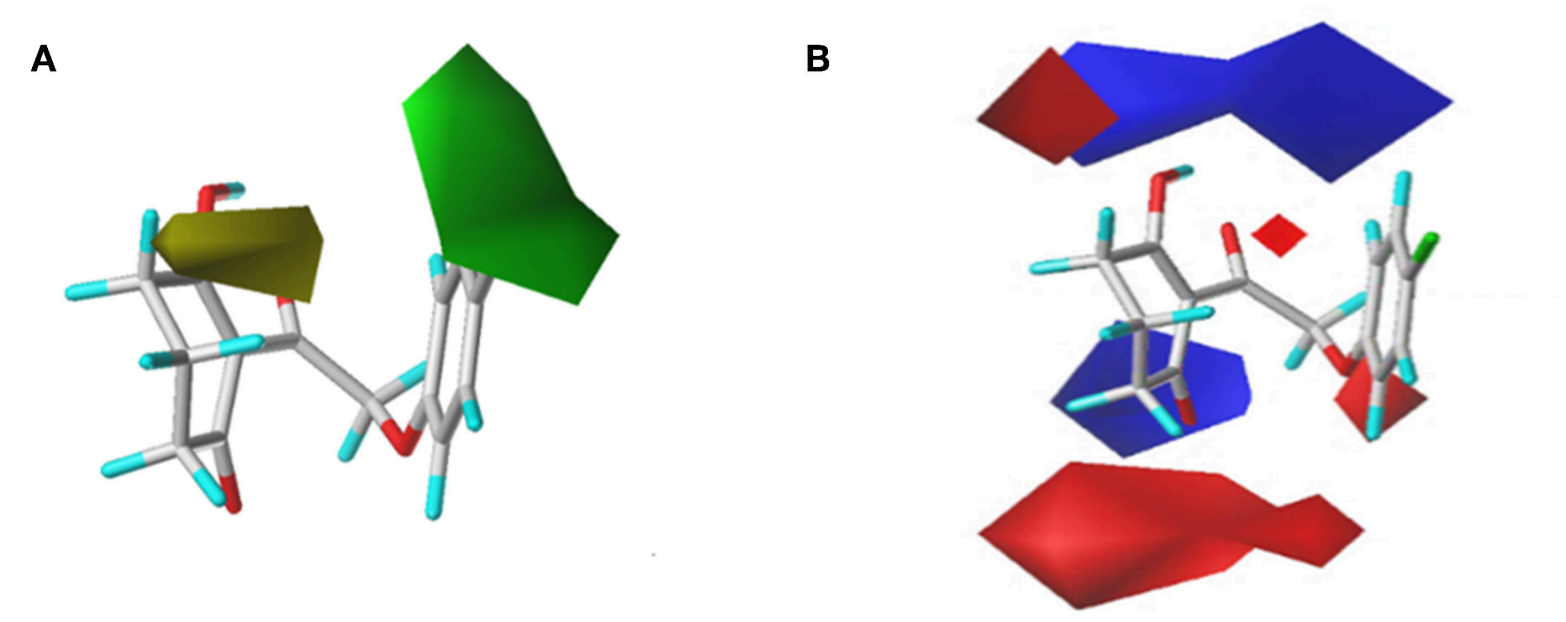
CoMFAStDev*Coeff contour maps based on the common framework. **(A)** Steric contour map. Green and yellow contours show regions where steric bulk has favorable and unfavorable effects on the inhibition ability, respectively. **(B)** Electrostatic contour map. Blue contours indicate regions where electro-positive groups increase the activity, while white contours indicate regions that were electro-negative.

In order to analyze the general feature of the steric and electrostatic field contribution, the structure–activity relationship calculation results of the CoMFA were demonstrated using the contour maps. The steric field result from common framework is shown in Figure 5A; the green color represented that the bulky group was favorable to the bioactivity of the HPPD inhibitors. On the contrary, the less bulky substituent, which was a benefit to the bioactivity, was marked in yellow. Comparing compound 01 with compound 06, it was found that the activity was increased with the change in p*K*_i_ values from 5.906 to 6.005 when the hydrogen atom at 4-position of the benzene ring was replaced by methyl. A small yellow area was in the near 5-position of the benzene ring, which suggested that bulky substituents at this site exerted an adverse impact on inhibition. For example, compounds containing a hydrogen atom always displayed better activity than the derivatives (comp. 02, 04, 10, 18, 25, 26, 29, 32, and 33) bearing one or two methyl groups as side chain. The contour map of the electrostatic descriptor based on the common framework is presented in Figure 5B, where the blue region indicated that the electropositive group was favorable to enhance the efficiency of the compounds; in contrast, the red region represented that the electronegative substituents would be conducive to the activity of the compounds. This map meant that the substituents at 2- and 3-position of R_2_ would have an electropositive effect, and it was better to have an optimum electronegative action at 4- and 6-position of R_1_.

**Figure 6:**
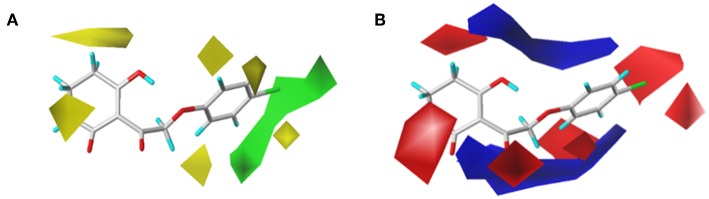
**(A)** Steric and **(B)** electrostatic contours of the CoMFA from molecular docking.

The CoMFAStDev^*^Coeff contour maps, based on molecular docking, are shown in Figure 6 and provided some additional guidance. The steric effects of the substituents need to be increased at the 4- and 5-position of R_2_, while the introduction of bulky groups should be avoided. The supplement offered by the electrostatic field was that the 4- and 6-position of R_2_ were more suitable for negative groups. For example, compound 42 (R_2_ = 2,4,6-tri-Cl, p*K*_i_ = 6.735) showed better activity than compounds 01 (R_2_ = H, p*K*_i_ = 5.906) and 09 (R_2_ = Cl, p*K*_i_ = 5.942).

### CoMSIA Results

**Figure 7:**
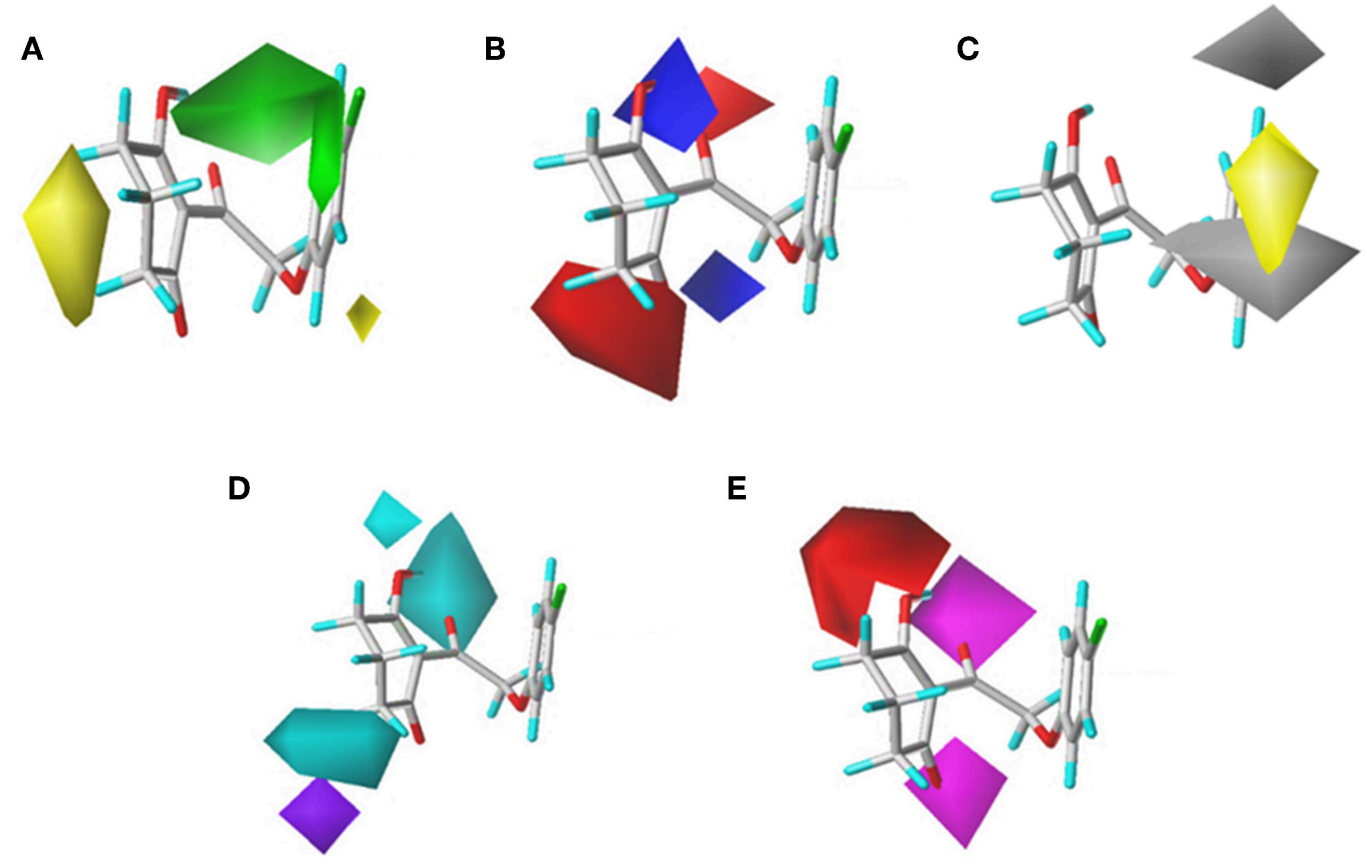
CoMSIAStDev*Coeff contour maps based on the common framework. **(A)** Steric contour map. **(B)** Electrostatic contour map. **(C)** Hydrophobic contour map. Yellow and white regions suggest the preference of hydrophobic groups and hydrophilic substitutions, respectively. **(D)** H-bond donor contour map. Cyan illustrates regions in which the introduction of a H-bond donor group is favored. Purple illustrates regions where the introduction of a H-bond donor group is disfavored. **(E)** H-bond acceptor contour map. Purple areas are the regions where H-bond acceptor is conducive to the activity; red areas are unfavorable.

To visualize the generated results, contour maps of CoMSIA based on the common framework is presented in Figure 7. The steric field and electrostatic field of the CoMSIA model based on the common framework (Figures 7A,B) provided the spatial and electrical impact of the substituents on the inhibitor, which were basically similar to the information obtained by the CoMFA contours. Figure 7C depicted the hydrophobic field of CoMSIA, in which white and yellow regions reflected the preference of hydrophilic substitutions and hydrophobic groups. Two white regions at the 3- and 5-position of R_2_ symbolized that the addition of the hydrophilic group would enhance the activity; however, introducing a hydrophobic group in the 4-position of R_2_ wrapped in yellow would also increase the inhibition, which was supported by compound 41 (R_2_ = 3,5-diF-4-CN, p*K*_i_ = 7.086) being more active than compound 24 (R_2_ = 3,5-diCl, p*K*_i_ = 6.020). The hydrogen bond donor is displayed in Figure 7D. In this plot, the cyan displayed positions where a H-bond donor group would be favorable for higher activity. In contrast, purple indicated positions where the H-bond donor of the target molecules is unfavored. There was a cyan contour near the 5-position of the six-membered ring and a small purple contour a little further from the 4-position. The content of Figure 7E showed the effect of the H-bond acceptor on the activity of the molecule, where the magenta and red contours stand for the promotion and suppression of inhibition effect, respectively. The characteristic contours were not at the substituent site, so we could infer that the influence of the H-bond acceptor was minimal to the activity of these series of compounds.

**Figure 8:**
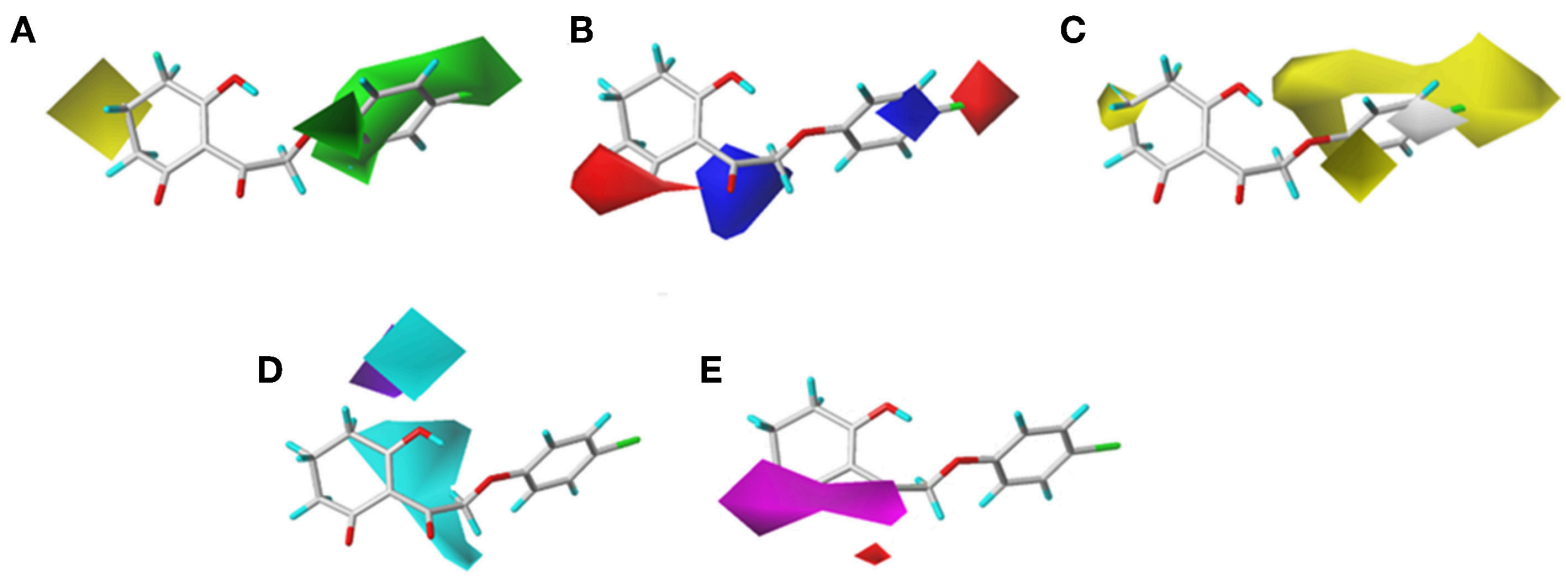
**(A)** Steric, **(B)** electrostatic, **(C)** hydrophobic, **(D)** H-bond donor, and **(E)** H-bond acceptor contours of the CoMSIA from molecular docking.

The CoMSIA results of molecular docking are shown in Figure 8, and the following discussion focused on the parts that were not obtained previously. The hydrophobic field of CoMSIA gave us a new perspective of a yellow contour with small size surrounding the 5-position of R_1_. It suggested that a hydrophobic substituent at this position would increase the inhibitory efficiency. The favorable results of hydrogen bond donors were formed around the hydroxyl group on the six-membered ring, while there were also favorable regions for hydrogen bond acceptors covering the ketone carbonyl of the triketone structure. These results were in line with the actual active data and could prove the accuracy and credibility of our CoMSIA model based on docking.

### Molecular Docking Analysis

**Figure 9:**
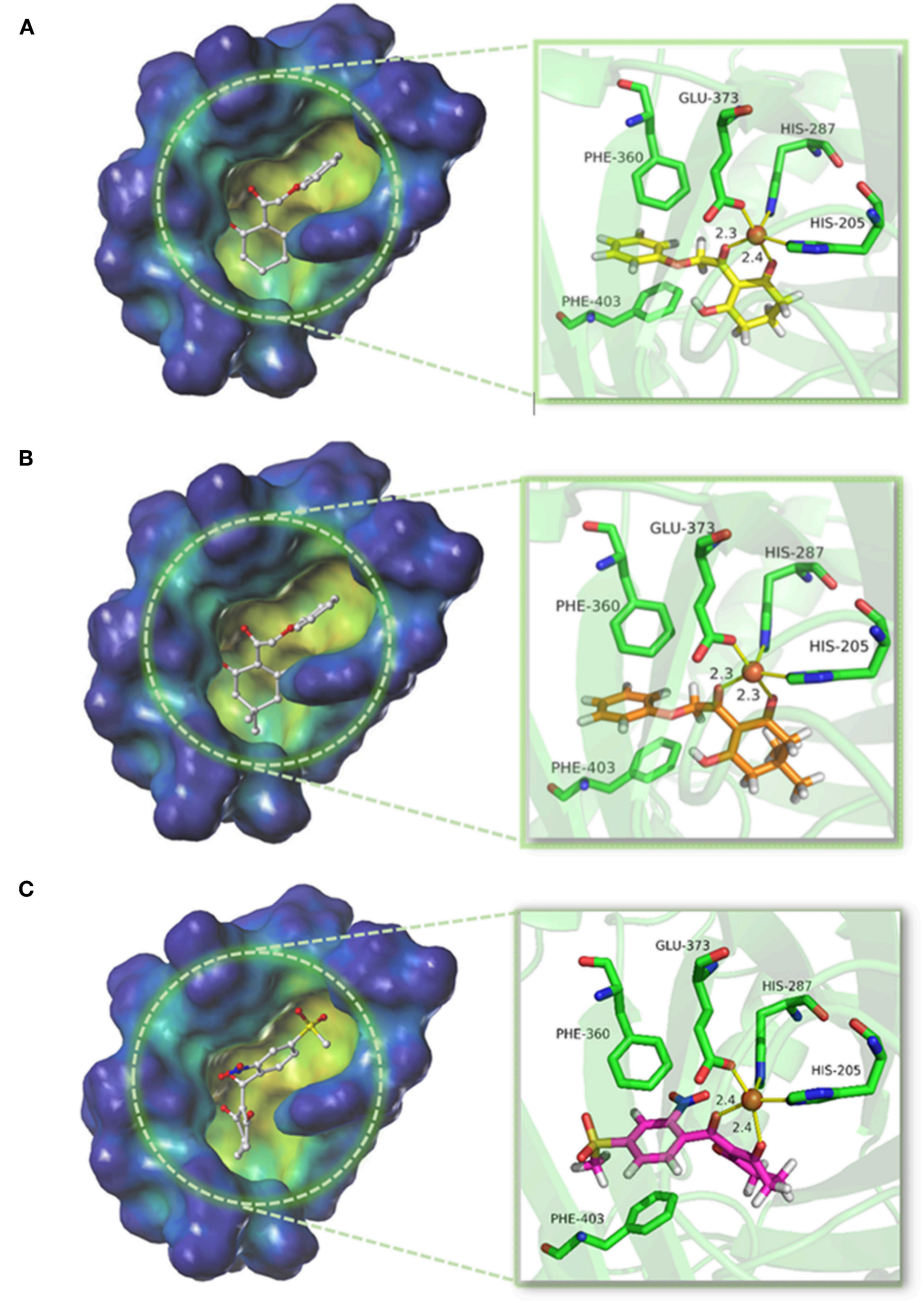
Binding model of **(A)** compound 01, **(B)** compound 02, and **(C)** mesotrione.

The structure of compounds 01 (p*K*_i_ = 5.906) and 02 (p*K*_i_ = 5.254) only differed from two methyl groups, but their activities were slightly different. Both were not as active as mesotrione (p*K*_i_ = 7.886), which aroused our interest. The overall orientation of these three molecules within the active site pocket of *At*HPPD is shown in Figure 9, and it was found that all molecules were fit well into the active cavity. In the process of complexing enzymes and inhibitors, the binding mode of the compound being studied was similar to that of the co-crystallized ligand (DAS869). The three amino acids (His205, His287, and Glu373) involved in chelation with the metal ion remain the same as the co-crystal complex (Yang et al., 2004). The two coordinating water molecules were displaced by different inhibitors. The distances from the 1,3-diketone moiety of the DAS869 inhibitor to the Fe(II) were measured to be 2.3 and 2.4 Å. The chelation distance of compounds 01 and 02 and mesotrione was refined to a range of 2.3–2.4 Å. It is worth noting that Phe360 and Phe403 formed π-π stacking interaction with the benzoyl moiety of DAS869, and similar effects occurred in the benzene of compounds 01 and 02 and mesotrione.

The conformations of the same part in compounds 01 and 02 were similar. Due to the presence of methyl, the activity of compound 02 was significantly weakened because the two methyls occupied a large pocket space. This inference was consistent with the QSAR results that on the 5-position of R_1_, the smaller group was beneficial to increase the inhibitor activity. The docking result of mesotrione showed that the conformation of the six-membered ring was different from that of compounds 01 and 02, and it fitted more closely to the active pocket. The activity of the compounds in this study was lower than that of mesotrione, probably because the oxygen atom in the framework structure affected the conformation of the molecule. The presence of an oxygen atom reversed the six-membered ring of compounds 01 and 02, which, although not affecting its coordination with the iron atom, reduced the activity of the inhibitor. At the same time, the carbon chain was elongated, causing the benzene ring to move back, and π-π interaction was weaker than that of mesotrione. To further explore the factors influencing activity, MD simulations were applied to these three compounds.

### MD Analysis

**Figure 10:**
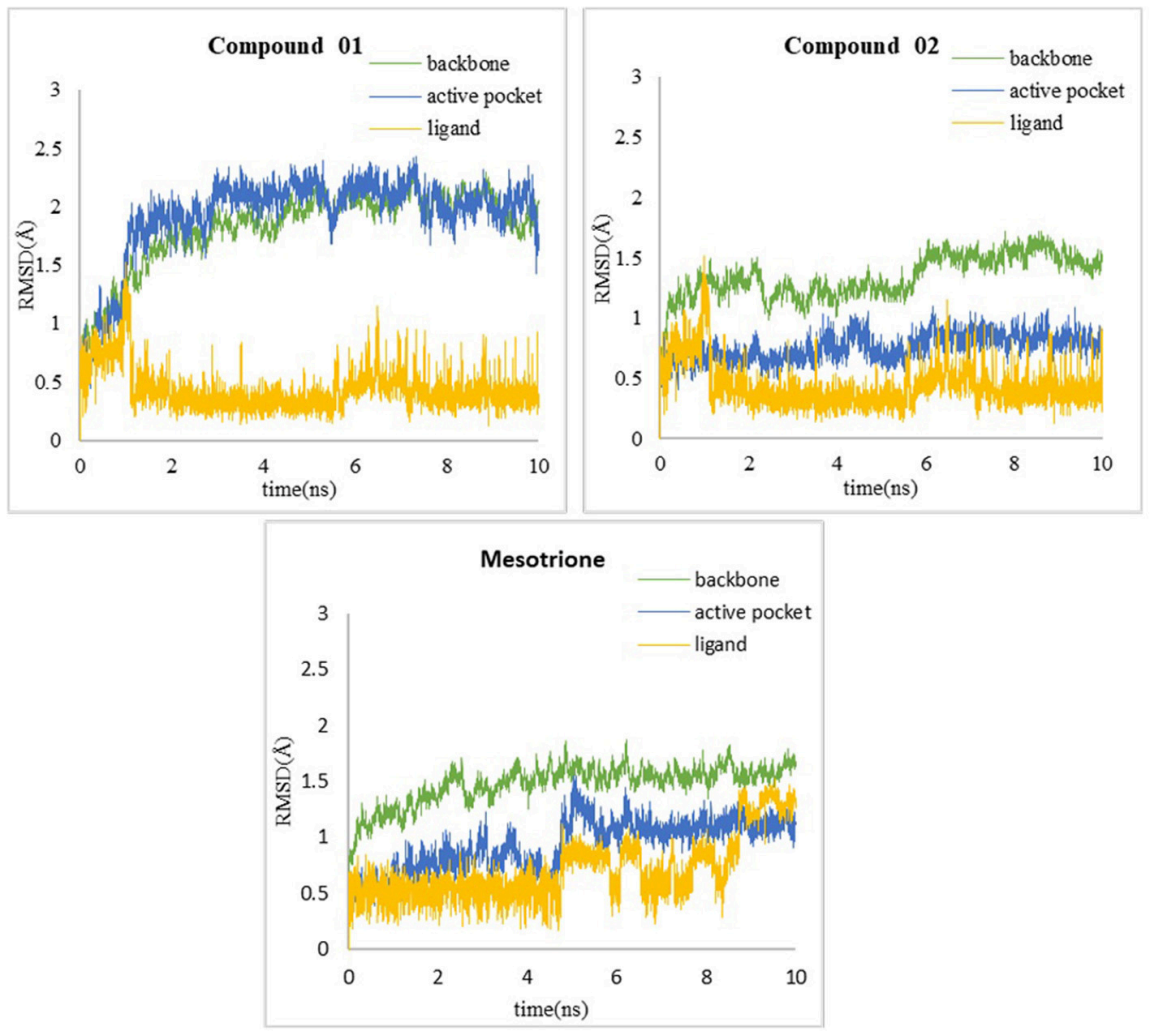
RMSD of **(A)** compound 01, **(B)** compound 02, and **(C)** mesotrione.

In order to verify whether the systems reached equilibrium during the dynamics simulation, the root-mean-square deviation (RMSD) was calculated, which reflected the dynamic change of the entire structure in the simulation process. RMSD values included the backbone Cα atoms of the protein, active pocket with residues of 5 Å around ligand, and the heavy atoms of ligand (Figure 10). All systems were dynamically changing throughout the kinetics. The RMSD values of the backbone of the compound 02 and mesotrione systems were small, showing higher stability throughout the simulation, and the skeleton structure of compound 01 was more unstable. It is worth noting that mesotrione in the protein complex was not as stable as ligands 01 and 02 during the simulation. Mesotrione entered an unstable phase at 5 ns and eventually restabilized at 9–10 ns. All the RMSDs were steady in the last 1-ns simulation process maintained within the 0.5-Å range. The equilibrium stage of the MD simulation was taken for the binding free energy and free energy decomposition analysis of each compound.

The calculated results are given in Table 3 including the van der Waals interaction energy (Δ*E*_vdw_), the electrostatic energy (Δ*E*_ele_), the polar solvation free energy (Δ*G*_PB_), the non-polar solvation free energy (Δ*G*_SA_), the interaction energy (Δ*E*_MM_), the solvation contribution (Δ*G*_sol_), and the overall binding free energy (Δ*G*_bind_). It could be seen that the total binding free energies of compounds 01 and 02 and mesotrione were −19.81, −3.65, and −28.34 kcal mol^−1^, respectively. The calculated binding energy was in good agreement with the experimental activity order. As shown in Table 3, the electrostatic terms occupied the principal driving forces for the three complexes, which made a supreme contribution to the binding free energy. The Δ*E*_vdw_, Δ*E*_ele_, and Δ*G*_SA_ calculated by the MM-PBSA approach were the favorable contributions to Δ*G*_bind_; in contrast, Δ*G*_PB_ had a certain passive effect. By comparing with systems 01 and 02, the addition of methyl groups at the 5-position of R_1_ led to significant distinction in each term and thus its herbicidal activity is poor. It was found that Δ*E*_ele_ of compound 02 (−76.36 kcal mol^−1^) was lower than that of compound 01 (−92.50 kcal mol^−1^). The unfavorable contribution, Δ*G*_PB_, of compound 02, which was 107.68 kcal mol^−1^, was stronger than that of compound 01, which was 102.45 kcal mol^−1^. Interestingly, the change in Δ*E*_vdw_ tended to increase the activity of compound 02, and Δ*G*_SA_ slightly increased, which had no impact on the overall trend. The Δ*E*_vdw_ contribution of mesotrione was significantly greater than 01 and 02, while the inhibition of Δ*G*_PB_ was also small; the contribution of Δ*E*_ele_ was similar to 02. The binding free energies (Δ*G*_exp_ = −*RT*ln *K*_*i*_) for the compounds were also calculated using the *K*_i_ values. The Δ*G*_exp_ of compounds 01 and 02 and mesotrione were −8.05, −7.16, and −10.74 kcal mol^−1^, respectively. We noted that MM-PBSA calculations systematically overvalued the binding free energies between ligand and protein for compound 01 and mesotrione systems. However, the value of Δ*G*_cal_ was qualitatively consistent with Δ*G*_exp_, confirming the reliability of MD simulation.

**Table 3:** Binding free energy (kcal mol^−1^) of compounds 01 and 02 and mesotrione^a^.

**System**	**Δ*E*_**vdw**_**	**Δ*E*_**ele**_**	**Δ*G*_**PB**_**	**Δ*G*_**SA**_**	**Δ*E*_**MM**_**	**Δ*G*_**sol**_**	**Δ*G*_**bind**_**
Compound 01	−26.48 (±2.49)	−92.50 (±6.88)	102.45 (±3.64)	−3.28 (±0.06)	−118.98 (±6.03)	99.17 (±3.63)	−19.81 (±4.72)
Compound 02	−31.30 (±3.13)	−76.36 (±5.52)	107.68 (±5.01)	−3.69 (±0.07)	−107.65 (±4.62)	104.00 (±4.95)	−3.65 (±4.57)
Mesotrione	−41.97 (±1.89)	−74.11 (±3.13)	91.38 (±3.01)	−3.64 (±0.05)	−116.08 (±3.72)	87.74 (±2.99)	−28.34 (±3.18)

a *ΔE_vdw_, van der Waals energy; ΔE_ele_, electrostatic energy; ΔG_PB_, polar solvation energy with the PB model; ΔG_SA_, non-polar solvation energy with the PB model; ΔE_MM_ = ΔE_vdw_ + ΔE_ele_, the interaction free energy; ΔG_sol_ = ΔG_PB_ + ΔG_SA_, the solvation free energy; ΔG_bind_ = ΔE_vdw_ + ΔE_ele_ + ΔG_PB_ + ΔG_SA_, the binding free energy. The number in the bracket indicates the standard error of the mean value*.

**Figure 11:**
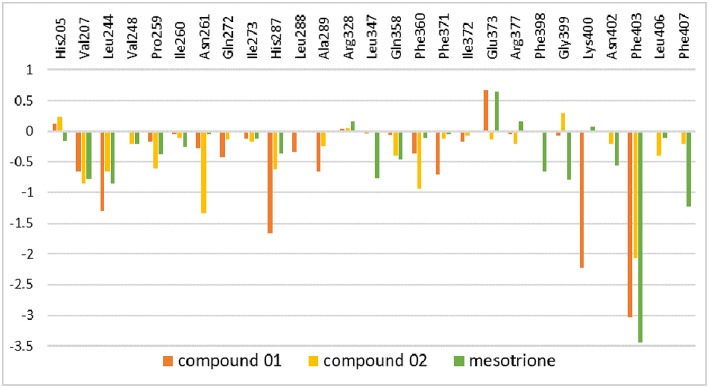
Inhibitor–residue interaction spectrum of complexes.

The amino residue contributions of HPPD binding with the ligand at the active site cavity are given in Figure 11. It was generated to understand the binding mechanism of protein–ligand. As listed in the plot, the residue groups including Val207, Leu244, His287, Ala289, Phe371, Glu373, Lys400, and Phe403 participated in the binding with molecule 01. Interestingly, the His205 and Glu373 produced a positive number combination of free energy, which was an unfavorable element even if they were involved in chelation with Fe(II). The candidates promoting contributions to the binding free energy in compound 02 were Val207, Leu244, Pro259, Asn261, His287, Phe360, and Phe403. The residue His205 played the same role as it did in system 01. Residues Val207, Leu244, Leu347, Phe398, Gly399, Phe403, and Phe407 made the greatest contribution to the binding energy for mesotrione, and Glu373 had a negative effect. The contribution of residue Phe403 to the binding of the three compounds obviously increased, which indicated the importance of π-π interaction between Phe403 and candidate ligands. Gly Leu llePhe Pro and Val, which belonged to the nonpolar amino acid family that contributed to nonpolar interactions, act as positive drivers of receptor–ligand binding. ArgAsnGln Glu His and Lys were polar amino acids, which was consistent with the conclusions of binding free energy and electrostatic played a major role in the interaction of molecules with protein.

## Conclusion

**Figure 12:**
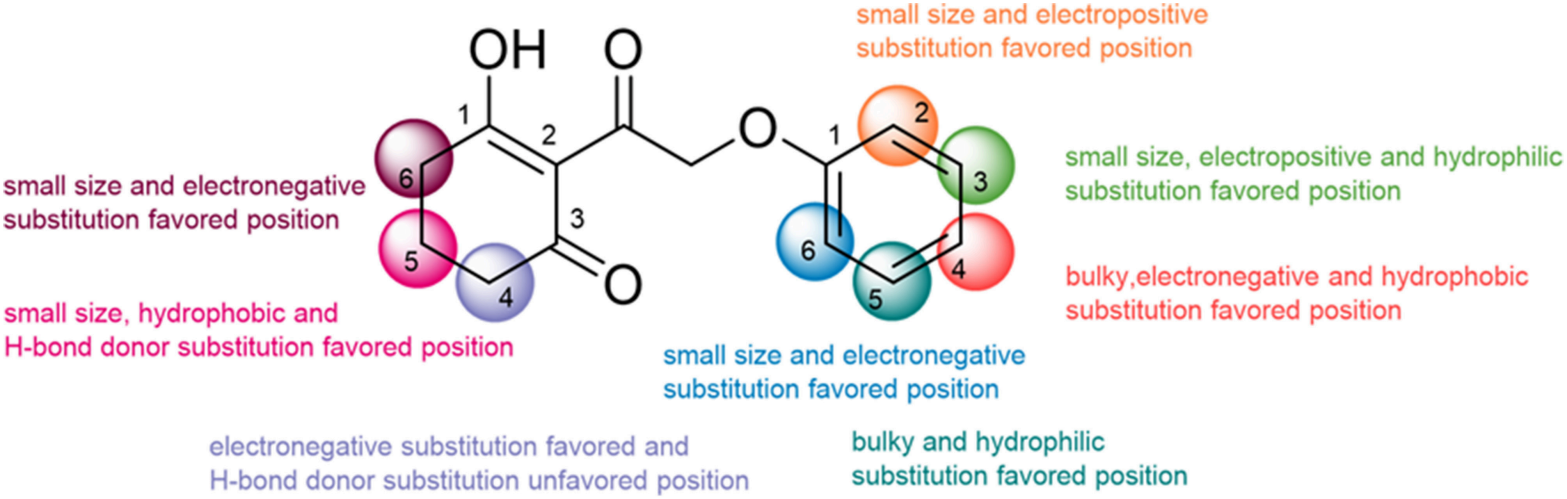
The summary of structure–activity relationship.

In the current work, 3D-QSAR models including CoMFA and CoMSIA with ideal cross-validated correlation coefficient values and best correlation coefficient values were established to analyze the 2-(aryloxyacetyl)cyclohexane-1,3-diones derivatives as valid HPPD inhibitors. The structural features conducive to enhance the activity are summarized in Figure 12. The methyl at the 5-position posed an adverse effect on the inhibitor by forming a steric hindrance as well as an effect of oxygen atom in the backbone on the molecular conformation, which was demonstrated by the result of molecular docking. The MD simulation and MM-PBSA energy calculation revealed that the electrostatic energy was the major driving force for ligand binding. It also illuminated the amino acid residues involved in inhibitor–HPPD interaction, in which the Phe403 was prominent in the systems. This study not only is helpful in clarifying the binding mechanism of the HPPD inhibitor but also provides useful information to the discovery of novel HPPD inhibitors.

## Author Contributions

YF and FY conceived and designed the workflow. Y-XL built 3D-QSAR models. K-HY performed molecular docking. M-QL and J-ZL developed the molecular dynamics. YF and Y-XL contributed to the discussion and analysis of the results. YF completed the manuscript. All authors read and approved the manuscript.

### Conflict of Interest Statement

The authors declare that the research was conducted in the absence of any commercial or financial relationships that could be construed as a potential conflict of interest.
